# Hypoglycemia and Cardiovascular Disease: Exploring the Connections

**DOI:** 10.7759/cureus.47784

**Published:** 2023-10-27

**Authors:** Kishan Akhaury, Anil Wanjari, Arya Harshyt Sinha, Mayank Kumar

**Affiliations:** 1 Medicine, Jawaharlal Nehru Medical College, Datta Meghe Institute of Higher Education and Research, Wardha, IND; 2 Anatomy, Jawaharlal Nehru Medical College, Datta Meghe Institute of Higher Education and Research, Wardha, IND; 3 Community Medicine, Jawaharlal Nehru Medical College, Datta Meghe Institute of Higher Education and Research, Wardha, IND

**Keywords:** peripheral arterial diseases, hypoglycemia, cardio vascular disease, diabetes, diabetes type 2

## Abstract

It has long been known that administering insulin or insulin secretagogues to treat diabetes has the unfavorable side effect of hypoglycemia. Because hypoglycemia can disrupt normal brain function, it can have a profound impact on people's lives. Studies have shown a connection between hypoglycemia and a higher risk of death and cardiovascular disease. Through experimental studies, numerous potential reasons for the start of cardiovascular events have been discovered. In addition, studies on people have demonstrated that hypoglycemia can result in ventricular arrhythmias. According to recent studies, a number of factors may affect the relationship between hypoglycemia, cardiovascular events, and mortality. Confounding factors may explain the apparent correlation, at least in part. People with comorbidities may experience more hypoglycemia, increasing their risk of mortality. Those who have type 1 or type 2 diabetes, however, seem to be more susceptible to the negative effects of hypoglycemia on the cardiovascular system. When choosing appropriate glucose-lowering treatments and setting glycemic objectives with patients, clinicians should be aware of this risk.

## Introduction and background

Cardiovascular diseases (CVDs) are the leading cause of death worldwide, killing more people annually than all other causes put together [1,2]. The WHO found that cardiovascular disease, which took the lives of an estimated one crore seventy-three lakh people worldwide in 2008, accounted for nearly 30 percent of all deaths. In addition, it is estimated that by 2030, approximately three crore people will die from cardiovascular diseases, mainly heart disease and stroke [2]. Of the total deaths reported in 2012, 67 percent were due to noncommunicable diseases, according to the WHO. More than 65 percent of all deaths occurred in low- and middle-income countries [3]. A remarkable discovery was that one crore people in their 70s currently suffer from non-communicable diseases. In addition, 80 percent of these deaths occurred in low- and middle-income countries [3]. Now, it is not true that CVD affects wealthy countries more than low- or middle-income countries. This may be due to the recent recognition of CVD in developing countries due to increased awareness of screening and the availability of diagnostic services. CVD accounts for most of the over two crore deaths each year caused by non-communicable diseases. Cancer is the second-leading cause of death from non-communicable diseases (NCDs), claiming more than one crore lives each year. Respiratory diseases are the third, with more than fifty lakh deaths, fifteen lakh of which are due to diabetes. It is said that 82 percent of all NCD cases can be traced to these four diseases [3].

## Review

Methodology

**Figure 1:**
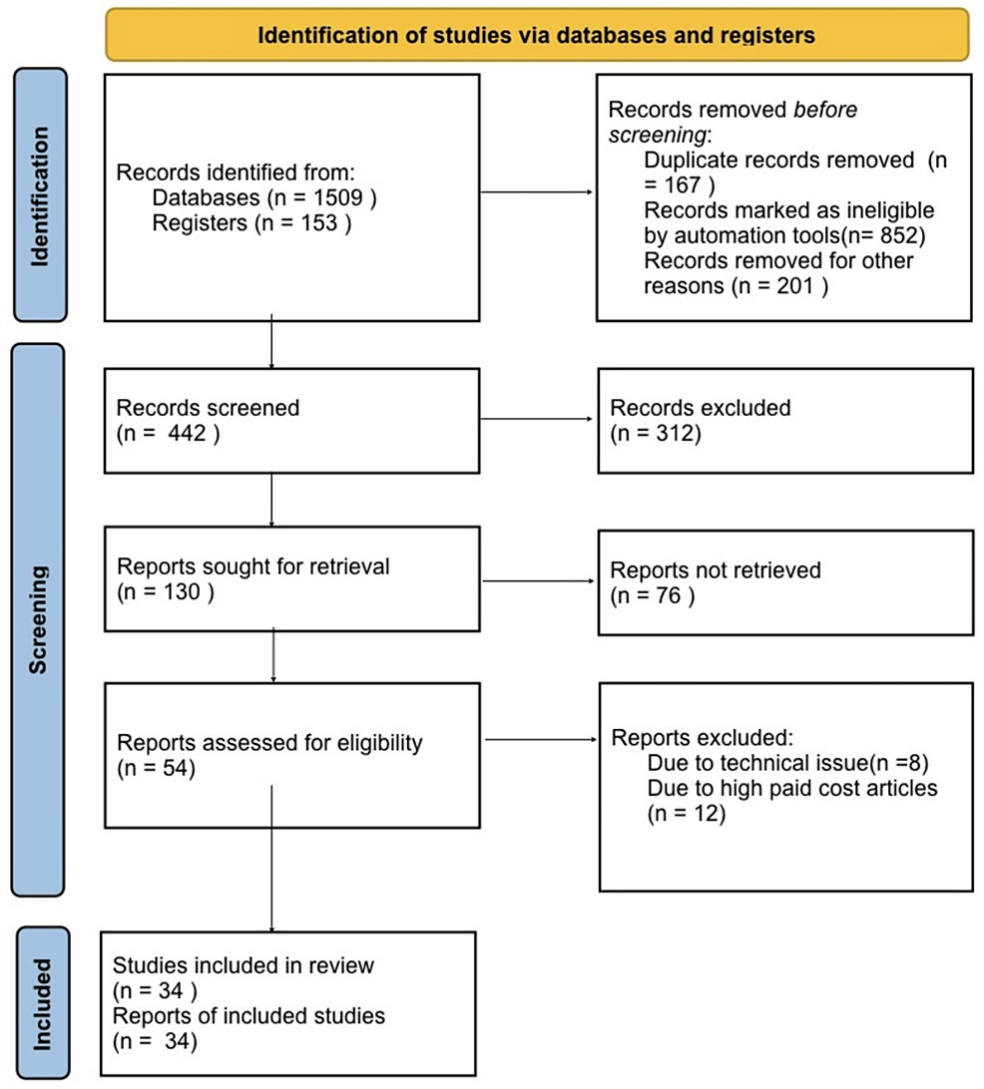
Search strategy utilised for this review

In conducting this narrative review, we initiated our search process by accessing a database like 'PubMed.' Our search terms encompassed key terms such as 'hypoglycemia,' 'diabetes,' 'cardiovascular disease,' and 'clinical techniques.' We specifically focused on English-language publications. In cases where multiple reports stemming from a single study were identified in the literature, we prioritized the most recent one. Our inclusion criteria were designed to incorporate only review papers that introduced fresh insights and findings. A visual representation of our search strategy can be found in Figure 1.

Risk factors for cardiovascular disease

Morbid Obesity

Excessive body fat, which is primarily connected to high levels of total fat intake, increased consumption of sugar-sweetened drinks, and a sedentary lifestyle, is the main risk factor for type 2 diabetes (T2D) [4,5]. It is crucial to remember that the body mass index (BMI) cutoff for Asians is lower than that for other ethnicities, often between 23 and 24 kg/m2. Over the course of their lives, a teenager who is obese at the age of 18 has a greater than 50 percent probability of having diabetes. The chance of this hazard increases to around 75 percent in an obese adolescent, with a BMI between 25 and 29.9 kg/m2 being considered "overweight" and a BMI of 30 kg/m2 or higher being termed "obese." Population studies [6] show that in the United States, over 35 percent of adults and 20 percent of teenagers are obese. This issue can be partly or completely attributed to children's general lack of physical activity. In this age range, 80 percent of girls and 75 percent of boys do not participate in the least amount of physical activity (150 minutes of moderate-intensity activity a week or 75 minutes of vigorous-intensity activity a week) that is advised. Furthermore, it's critical to keep in mind that, when compared to low-income countries, high-income countries have the highest rate of physical inactivity [1]. Overweight or obese people make up at least 70 percent of T2D patients [7]. Cardiovascular mortality in this group of people rises by up to 45 percent for every 5-unit increase in BMI beyond the threshold of 25 [8]. According to the study, adults with BMIs between 30 and 35 kg/m2 have a shorter estimated median lifespan (40 years) compared to those with BMIs below 30 kg/m2. Similarly, those with BMIs between 40 and 45 kg/m2 are reported to have a median survival rate that is 8 to 10 years lower than individuals with a BMI below 40 kg/m2 [8].

Hypertension

The risk of cardiovascular disease is increased when hypertension and diabetes mellitus coexist frequently. According to the United Kingdom Prospective Diabetes Study (UKPDS 38), people with carefully regulated blood pressure had a 35 percent decreased risk of myocardial infarction, sudden cardiac death, stroke, and peripheral vascular disease. In comparison to those whose blood pressure was not properly managed, this group had a 40 percent decreased risk of developing retinopathy requiring photocoagulation, vitreous hemorrhage, and fatal or nonfatal renal failure [9]. Similar conclusions emerged from the ACCORD trial's secondary analysis, which showed that tight blood pressure control significantly decreased major cardiovascular events by 26 percent [9,10]. According to the most recent available data, the American Heart Association/American College of Cardiology (AHA/ACC) advises starting lifestyle changes and taking antihypertensive medication when your blood pressure reaches or exceeds 130/80 mmHg. The aim of therapy should be to lower the patient's blood pressure to less than 130/80 mmHg if this can be done without resulting in hypotension or syncope [11,12].

Disorder of Lipoproteins

Abnormal lipid levels are more prevalent in people with T2D, which raises their risk of cardiovascular disease. Cholesterol-lowering medications can reduce the risk of atherosclerotic vascular events and diabetes-related mortality in both primary and secondary prevention settings, according to important studies [13-15]. According to meta-analyses of statin drugs, there is a proportional reduction of 10 percent in overall mortality and 15 percent in vascular mortality for every mmol/l (39 mg/dl) decrease in low-density lipoprotein (LDL) cholesterol [16]. A new diabetes diagnosis in statin users had an odds ratio (OR) of 1.09 in a meta-analysis of 13 randomized statin trials with 91,140 individuals. According to this, using statins for four years would cause one more case of diabetes in 255 people while averting 5,4 cardiovascular events. All people with prediabetes or diabetes who are 40 years of age or older should take statins into consideration. Prior to starting medication, diabetic patients 75 years of age and older should consider the advantages and disadvantages of statins. Statin users had a decreased risk of all-cause mortality compared to nonusers in a population-based Icelandic study of men and women aged 66 to 96 years, with a mean age of 76 years (hazard ratio: 0.47; 95 percent confidence interval: 0.32 to 0.71). Glycemic management is likewise crucial.

Cigarette Smoking

The risk of T2D and diabetic complications is considerably increased by tobacco use [1,17]. The most vulnerable category consists of regular smokers. Studies on diabetics have also shown an increased risk of heart disease, capillary issues, and early death due to exposure to secondhand smoke. However, microalbuminuria, hypertension, cholesterol, and insulin resistance all dramatically declined after stopping smoking. A doctor should assess their patients' blood sugar levels and think about diabetes treatment once they have determined all the risk factors for diabetes and cardiovascular disease.

Cardiovascular effects of hypoglycaemia

A rising amount of research indicates a link between hypoglycemia, the beginning of heart issues, and early mortality. The database includes case reports detailing various cardiac arrhythmias brought on by hypoglycemia, in addition to research exhibiting aberrant cardiac repolarization. Most clinical investigations have used type 1 diabetics or those without the disease as their subjects. Patients with type 2 diabetes, however, have not been the focus of a lot of studies [18]. The production of catecholamines increases as a result of sympathoadrenal system activation brought on by hypoglycemia. Hemodynamics and hemorheology are significantly influenced by these catecholamines. Myocardial contractility, heart rate, and cardiac output all quickly increase when sympathetic stimulation is engaged. Another result of the major blood vessels greater flexibility is a decrease in central systolic pressure [19,20]. Plasma potassium levels may suddenly drop [21], and this could lead to electrophysiological and electrocardiographic abnormalities, which could lead to faulty cardiac conduction and repolarization [21]. Additionally, hypoglycemia produces sudden changes in inflammatory indicators, blood coagulation, cellular adhesion, and endothelial dysfunction. By reducing endothelial cells' ability to conduct blood flow, obstructing tissue perfusion, and suppressing blood clotting, the aforementioned behaviors raise the risk of intravascular coagulation and thrombosis [22].

Non-diabetic people's autonomic reactions to cardiac stress are slowed down and decreased when they have previously experienced hypoglycemia. This momentary reduction in autonomic cardiac responses might have an impact on how responsive the heart is to other stimuli [23]. It has been shown that people with type 2 diabetes exhibit hemorrhagic and inflammatory reactions to hypoglycemia that last for a protracted period of time. Functional issues that continue even after blood sugar levels have stabilized can lead to blood vessel thrombosis [24]. Contrary to expectations, individuals getting conventional care and having greater HbA1c concentrations also had a higher death risk from cardiovascular events brought on by hypoglycemia, according to the ACCORD Trial [14]. In order to prevent recurrence, repeated hypoglycemia may reduce the sympathetic-adrenal response. However, it is not yet known whether such exposure raises the risk of cardiovascular issues [23].

In people with type 2 diabetes, a significant portion of whom have premature cardiovascular disease, the brief changes in blood flow dynamics that take place during episodes of hypoglycemia have the potential to cause acute cardiovascular events like myocardial ischemia and infarction, cardiac failure, and cardiac arrhythmias [23]. Numerous occurrences of acute cardiovascular events have been associated with hypoglycemia. Additionally, research has demonstrated that ischemia-related ECG alterations can happen both with and without angina [25].

Clinical techniques

Psychological Difficulties

The negative consequences of hypoglycemia, which include a range of unpleasant symptoms and potential hazards, usually cause patients and their families to develop a phobia of hypoglycemia. The detrimental effects of hypoglycemia anxiety on people's general quality of life in a variety of nations and cultures have been adequately documented by numerous studies [26,27]. An elevated fear of hypoglycemia is generally linked to a history of severe hypoglycemic episodes, particularly those that resulted in bodily harm, accidents, social shame, unconsciousness, or cognitive impairment. People who are more susceptible to severe hypoglycemia episodes due to a lack of situational awareness have a strong tendency to feel nervous about them. But it's important to note that, paradoxically, about one-third of these people score poorly on exams meant to gauge their fear of hypoglycemia [27]. Hypoglycemia, especially nocturnal episodes, is more likely to cause parents of children with type 1 diabetes anxiety. These parents are under a great deal of emotional stress as a result of this phenomenon [28].

The management and control of diabetes may be severely impacted by anxiety brought on by hypoglycemia. Some people may use a variety of strategies to control their anxiety and lower their risk of hypoglycemia. The deliberate maintenance of increased blood glucose levels, which leads to prolonged hyperglycemia, is one such tactic. Numerous studies have connected heightened levels of anxiety due to hypoglycemia to poor metabolic regulation, as seen by more frequently occurring hyperglycemic blood glucose readings and higher HbA1c values [28]. Although some patients may be hesitant to tell their doctors that they have severe hypoglycemia, it is important to investigate these circumstances in a clinical context. It is crucial to discuss components of the incident that are probably connected to psychological effects on patients and their families, in addition to examining criteria like etiology, frequency, and severity. Influences on an episode include external circumstances, interpersonal effects, and emotional effects. Situational characteristics define the circumstances of the incident, such as whether it takes place in a crowded area or when the victim is alone. The event's social effects include the presence of friends or coworkers. The individual's level of anxiety or sense of danger is taken into account when determining the emotional effects. It is critical to assess the changes made by patients or members of their families to their diabetes care routines and practices after a hypoglycemia episode. The unusual but well-known phenomenon known as the "dead-in-bed condition" presents a unique problem and ethical dilemma for healthcare providers when it comes to educating patients and their families about hypoglycemia. The potential consequences of mortality from hypoglycemia on the relatives of people who have this condition have received very little research. The mortality risk of severe nocturnal hypoglycemia, however, worries a lot of patients and their families, especially if they have themselves had a hypoglycemic seizure [29]. Anecdotal evidence suggests that healthcare professionals regularly fail to alert patients and their families to the risks of deadly ventricular arrhythmias brought on by hypoglycemia. The psychological impacts experienced by patients' families are, however, the subject of few scientific investigations. Even though hypoglycemia at night is widespread, fatal occurrences are incredibly rare. Therefore, suggesting it as a prospective result could lead to unjustified fear. Additional study is necessary to completely understand the risk factors and mechanisms connected to fatal nocturnal hypoglycemia. This knowledge would be crucial for directing the creation of suitable patient education resources and recommendations.

Basic Clinical Techniques

Sulfonylurea and insulin prescribers are required to alert their patients to the possibility of hypoglycemia when using these drugs. Referring these patients to diabetic education programs in a clinical facility with a large patient volume is advised so they can get complete instructions on identifying, avoiding, and treating hypoglycemic episodes. All medical practitioners should have the crucial clinical skill of being able to identify hypoglycemia in patients who are not aware of it. The questions from the ADA Hypoglycemia Working Group [1] can be used as a reference to ensure that a comprehensive review is carried out following each visit. The International Hypoglycemia Study Group has also created educational resources to help doctors and patients better understand the challenges of managing diabetes [30].

New methods for lowering the occurrence of hypoglycemia in diabetic patients have been developed as a result of recent developments in the field of diabetes therapy [27]. Patients with type 1 and type 2 diabetes who require insulin may experience less hypoglycemia when taking long-acting basal insulin, such as insulin degludec. Studies [27-29] have shown that insulin degludec is more efficient than insulin glargine U100 at lowering the incidence of severe hypoglycemia in patients with both forms of diabetes. An insulin pump may be helpful for people with type 1 diabetes who are more prone to hypoglycemia [31]. According to a study, the frequency of hypoglycemic episodes can also be greatly decreased by integrating continuous glucose monitoring (CGM) in real-time into a well-established insulin treatment plan [32]. People with type 1 diabetes who use a hybrid closed-loop pump or a threshold-suspend pump have less hypoglycemia while maintaining proper glycemic control, according to research [33]. People who frequently experience severe hypoglycemia despite continued efforts to prevent it and those with a decreased ability to recognize hypoglycemia may be candidates for islet transplantation [34]. The goal of this study is to look into ways to lower the risk of hypoglycemia in people with a history of cardiovascular disease. According to the ADA's management recommendations, individuals with clinically severe hypoglycemia or little awareness of hypoglycemia should have their glycemic goals altered [34]. Patients taking sulfonylureas or insulin should have their cardiovascular comorbidities watched more attentively. It may be required to raise glucose targets and, if necessary, HbA1c objectives to 55 mmol/mol (7 percent), in order to prevent hypoglycemia in vulnerable individuals. By switching to a treatment regimen that excludes the use of sulfonylureas and insulin, type 2 diabetics may be able to lower their risk of hypoglycemia. Increased amounts of glycated hemoglobin may also follow this alternate method [34]. Modern technology like pumps, continuous glucose monitoring (CGM), and insulin analogues may help decrease hypoglycemia. A correlation between the drop in hypoglycemia rates and a lower risk of cardiovascular disease or a longer life from diabetes cannot, however, be made due to a lack of evidence. The fact that the trials were conducted over a relatively short period of time and that the participants' baseline cardiovascular risk levels were relatively low may be to blame for the lack of evidence.

Findings from the different studies are included in Table 1.

**Table 1:** Findings of studies included in this review article

Author Name	Study Title	Findings
Imamura F, O’Connor L, Ye Z, et al. [5]	Consumption of sugar-sweetened beverages, artificially sweetened beverages, and fruit juice and incidence of type 2 diabetes	Presents a systematic review and meta-analysis on beverage consumption and the incidence of type 2 diabetes.
Sluik D, Boeing H, Montonen J, et al. [7]	Associations between general and abdominal adiposity and mortality in individuals with diabetes mellitus	Explores associations between adiposity and mortality in individuals with diabetes.
Whitlock G, Lewington S, Sherliker P, et al. [8]	Body-mass index and cause-specific mortality in 900,000 adults: collaborative analyses of 57 prospective studies	Examines the relationship between body-mass index and cause-specific mortality.
Leon B, Maddox T [11]	Diabetes and cardiovascular disease: epidemiology, biological mechanisms, treatment recommendations and future research	Discusses the relationship between diabetes and cardiovascular disease.
Whelton PK, Carey RM, Aronow WS, et al. [12]	ACC/AHA/AAPA/ABC/ACPM/AGS/APhA/ASH/ASPC/NMA/PCNA guideline for the prevention, detection, evaluation, and management of high blood pressure in adults	Provides guidelines for the management of high blood pressure.
Taylor F, Huffman MD, Macedo AF, et al. [13]	Statins for the primary prevention of cardiovascular disease	Investigates the use of statins for primary prevention of cardiovascular disease.
Sattar N, Preiss D, Murray H, et al. [18]	Statins and risk of incident diabetes: a collaborative meta-analysis of randomised statin trials	Analyzes the risk of incident diabetes associated with statin use.
Fisher BM, Gillen G, Hepburn DA, Dargie HJ, Frier BM [20]	Cardiac responses to acute insulin-induced hypoglycemia in humans	Investigates cardiac responses to acute insulin-induced hypoglycemia in humans.
Petersen KG, Schlüter KJ, Kerp L [22]	Regulation of serum potassium during insulin-induced hypoglycemia	Examines the regulation of serum potassium during insulin-induced hypoglycemia.
Frier BM, Schernthaner G, Heller SR [23]	Hypoglycemia and cardiovascular risks	Discusses the cardiovascular risks associated with hypoglycemia.
Wright RJ, Newby DE, Stirling D, Ludlam CA, Macdonald IA, Frier BM [24]	Effects of acute insulin-induced hypoglycemia on indices of inflammation: putative mechanism for aggravating vascular disease in diabetes	Investigates the effects of acute insulin-induced hypoglycemia on inflammation and its potential role in aggravating vascular disease.
Adler GK, Bonyhay I, Failing H, Waring E, Dotson S, Freeman R [25]	Antecedent hypoglycemia impairs autonomic cardiovascular function: implications for rigorous glycemic control	Analyzes the impact of antecedent hypoglycemia on autonomic cardiovascular function and its implications for glycemic control.
Chow E, Iqbal A, Walkinshaw E, et al. [26]	Prolonged prothrombotic effects of antecedent hypoglycemia in individuals with type 2 diabetes	Investigates the prothrombotic effects of prolonged antecedent hypoglycemia in individuals with type 2 diabetes.
Wright RJ, Frier BM [27]	Vascular disease and diabetes: is hypoglycemia an aggravating factor?	Explores the relationship between vascular disease, diabetes, and hypoglycemia.
Hendrieckx C, Halliday JA, Bowden JP, et al. [29]	Severe hypoglycemia and its association with psychological well-being in Australian adults with type 1 diabetes attending specialist tertiary clinics	Investigates the impact of severe hypoglycemia on psychological well-being in Australian adults with type 1 diabetes.

## Conclusions

In conclusion, the global rise in noncommunicable diseases (NCDs), particularly cardiovascular diseases (CVDs), presents a significant public health challenge. The increasing prevalence of NCDs, combined with their staggering impact on mortality rates, underscores the urgent need for effective management and prevention strategies. This review has shed light on the intricate relationship between diabetes, hypoglycemia, and cardiovascular events, emphasizing that hypoglycemia can exert a detrimental influence on the cardiovascular system. It is evident that individuals with diabetes, particularly those with comorbidities, are more susceptible to the adverse effects of hypoglycemia, potentially leading to cardiovascular events, arrhythmias, and increased mortality. As such, it is imperative for clinicians to consider the risk of hypoglycemia when choosing glucose-lowering treatments and setting glycemic targets with their patients. Moreover, identifying and addressing the risk factors for diabetes and CVD, such as morbid obesity, hypertension, and lipid disorders, remains crucial in the fight against these diseases.

Furthermore, this review underscores the importance of not only clinical techniques for recognizing and managing hypoglycemia but also addressing the psychological difficulties that arise from the fear of hypoglycemia. Patients' anxiety and avoidance behaviors can significantly impact their diabetes management and overall quality of life. Healthcare providers must take these psychological aspects into account when devising treatment plans and educating patients. Recent advancements in diabetes therapy, such as insulin analogues and continuous glucose monitoring, offer promising avenues to mitigate hypoglycemia risk, particularly in patients with a history of cardiovascular disease. However, further research is needed to better understand the long-term impacts of these interventions on cardiovascular outcomes. In this ever-evolving landscape of diabetes and cardiovascular care, a multidisciplinary approach that combines medical, psychological, and technological strategies is essential to reduce the burden of these noncommunicable diseases on a global scale.

## References

[1] (2023). Global Status Report on Noncommunicable Diseases. https://apps.who.int/iris/bitstream/handle/10665/148114/9789241564854_eng.pdf?sequence=1&isAllowed=y.

[2] (2023). WHO Updates on Cardiovascular Disease. https://www.who.int/health-topics/cardiovascular-diseases#tab=tab_1.

[3] (2023). Noncommunicable Diseases. https://www.who.int/en/news-room/fact-sheets/detail/noncommunicable-diseases.

[4] Dabelea D (2009). The accelerating epidemic of childhood diabetes. Lancet.

[5] Imamura F, O'Connor L, Ye Z, Mursu J, Hayashino Y, Bhupathiraju SN, Forouhi NG (2015). Consumption of sugar sweetened beverages, artificially sweetened beverages, and fruit juice and incidence of type 2 diabetes: systematic review, meta-analysis, and estimation of population attributable fraction. BMJ.

[6] Hales C, Carroll M, Fryar C, Ogden C (2017). Prevalence of obesity among adults and youth: United States, 2015-2016.. NCHS data br.

[7] Sluik D, Boeing H, Montonen J (2011). Associations between general and abdominal adiposity and mortality in individuals with diabetes mellitus. Am J Epidemiol.

[8] Whitlock G, Lewington S, Sherliker P (2009). Body-mass index and cause-specific mortality in 900 000 adults: collaborative analyses of 57 prospective studies. Lancet.

[9] UK Prospective Diabetes Study Group (1998). Tight blood pressure control and risk of macrovascular and microvascular complications in type 2 diabetes: UKPDS 38. BMJ.

[10] Cushman WC, Evans GW, Byington RP (2010). Effects of intensive blood-pressure control in type 2 diabetes mellitus. N Engl J Med.

[11] Leon B, Maddox T (2015). Diabetes and cardiovascular disease: epidemiology, biological mechanisms, treatment recommendations and future research. World J. Diabetes.

[12] Whelton PK, Carey RM, Aronow WS (2018). 2017 ACC/AHA/AAPA/ABC/ACPM/AGS/APhA/ASH/ASPC/NMA/PCNA guideline for the prevention, detection, evaluation, and management of high blood pressure in adults: executive summary: a report of the American College of Cardiology/American Heart Association Task Force on clinical practice guidelines. Hypertension.

[13] Taylor F, Huffman MD, Macedo AF (2013). Statins for the primary prevention of cardiovascular disease. Cochrane Database Syst. Rev. 1.

[14] Cholesterol Treatment Trialists’ (CTT) Collaborators (2005). Efficacy and safety of cholesterol-lowering treatment: prospective meta-analysis of data from 90,056 participants in 14 randomised trials of statins. Lancet.

[15] Heart Protection Study Collaborative Group (2013). MRC/BHF heart protection study of cholesterol lowering with simvastatin in 5963 people with diabetes: a randomised placebo-controlled trial. Lancet.

[16] Kearney PM, Blackwell L, Collins R (2008). Cholesterol treatment trialists’ collaborators, efficacy of cholesterol-lowering therapy in 18,686 people with diabetes in 14 randomised trials of statins: a meta-analysis. Lancet.

[17] Colhoun H, Betteridge D, Durrington P (2004). Primary prevention of cardiovascular disease with atorvastatin in type 2 diabetes in the Collaborative Atorvastatin Diabetes Study (CARDS): multicentre randomized placebo-controlled trial. Lancet.

[18] Sattar N, Preiss D, Murray H (2010). Statins and risk of incident diabetes: a collaborative meta-analysis of randomised statin trials. Lancet.

[19] Olafsdottir E, Aspelund T, Sigurdsson G (2011). Effects of statin medication on mortality risk associated with type 2 diabetes in older persons: the population-based AGES-Reykjavik Study. BMJ Open.

[20] Fisher BM, Gillen G, Hepburn DA, Dargie HJ, Frier BM (1990). Cardiac responses to acute insulin-induced hypoglycemia in humans. Am J Physiol.

[21] Sommerfield AJ, Wilkinson IB, Webb DJ, Frier BM (2007). Vessel wall stiffness in type 1 diabetes and the central hemodynamic effects of acute hypoglycemia. Am J Physiol Endocrinol Metab.

[22] Petersen KG, Schlüter KJ, Kerp L (1982). Regulation of serum potassium during insulin-induced hypoglycemia. Diabetes.

[23] Frier BM, Schernthaner G, Heller SR (2011). Hypoglycemia and cardiovascular risks. Diabetes Care.

[24] Wright RJ, Newby DE, Stirling D, Ludlam CA, Macdonald IA, Frier BM (2010). Effects of acute insulin-induced hypoglycemia on indices of inflammation: putative mechanism for aggravating vascular disease in diabetes. Diabetes Care.

[25] Adler GK, Bonyhay I, Failing H, Waring E, Dotson S, Freeman R (2009). Antecedent hypoglycemia impairs autonomic cardiovascular function: implications for rigorous glycemic control. Diabetes.

[26] Chow E, Iqbal A, Walkinshaw E (2018). Prolonged prothrombotic effects of antecedent hypoglycemia in individuals with type 2 diabetes. Diabetes Care.

[27] Wright RJ, Frier BM (2008). Vascular disease and diabetes: is hypoglycaemia an aggravating factor?. Diabetes Metab Res Rev.

[28] Desouza C, Salazar H, Cheong B, Murgo J, Fonseca V (2003). Association of hypoglycemia and cardiac ischemia: a study based on continuous monitoring. Diabetes Care.

[29] Hendrieckx C, Halliday JA, Bowden JP, Colman PG, Cohen N, Jenkins A, Speight J (2014). Severe hypoglycaemia and its association with psychological well-being in Australian adults with type 1 diabetes attending specialist tertiary clinics. Diabetes Res Clin Pract.

[30] Evans M, Khunti K, Mamdani M, Galbo-Jørgensen CB, Gundgaard J, Bøgelund M, Harris S (2013). Health-related quality of life associated with daytime and nocturnal hypoglycaemic events: a time trade-off survey in five countries. Health Qual Life Outcomes.

[31] Gonder-Frederick L, Nyer M, Shepard JA, Vajda K, Clarke W (2011). Assessing fear of hypoglycemia in children with type 1 diabetes and their parents. Diabetes Manag (Lond).

[32] Anderbro T, Gonder-Frederick L, Bolinder J (2015). Fear of hypoglycemia: relationship to hypoglycemic risk and psychological factors. Acta Diabetol.

[33] Driscoll KA, Raymond J, Naranjo D, Patton SR (2016). Fear of hypoglycemia in children and adolescents and their parents with type 1 diabetes. Curr Diab Rep.

[34] Gonder-Frederick LA, Vajda KA, Schmidt KM (2013). Examining the behaviour subscale of the hypoglycaemia fear survey: an international study. Diabet Med.

